# Accelerated identification of serine racemase inhibitor from *Centella asiatica*

**DOI:** 10.1038/s41598-020-61494-1

**Published:** 2020-03-13

**Authors:** Komal Rani, Mitali Tyagi, Mohit Mazumder, Akanksha Singh, Annaian Shanmugam, Krishna Dalal, Manoj Pillai, Gourinath Samudrala, Saroj Kumar, Alagiri Srinivasan

**Affiliations:** 1 Department of Biophysics, All India Institute of Medical Sciences, New Delhi, India; 2 School of Life Sciences, Jawaharlal Nehru University, New Delhi, India; 3 Sciex, Gurgaon, India; 4 Centre of Advanced Study in Marine Biology, Faculty of Marine Sciences, Annamalai University, Parangipettai, India; 5 All India Institute of Ayurveda, New Delhi, India; 6 Present Address: Department of Biochemistry, Jamia Hamdard, Hamdard Nagar, New Delhi, India

**Keywords:** Drug screening, Neurological disorders

## Abstract

Serine racemase (SR) converts the free form of L-serine into D-serine (DS) in the mammalian brain. The DS functions as a co-agonist of N-methyl D-aspartate (NMDA) receptor. The over- activation of NMDA receptor leads to many neurological disorders like stroke, amyotrophic lateral sclerosis, Alzheimer’s disease and an effective inhibitor of SR could be a corrective method for the receptor over-activation. We report for the first time here a rapid way of purifying and identifying an inhibitor from medicinal plants known to have the neuro-protective effect. We have purified SR inhibitor from the methanolic extract of *Centella asiatica* by affinity method. High resolution mass spectrometry and infrared spectroscopy were used to identify the ligand to be madecassoside. We have shown the madecassoside binding *in silico* and its inhibition of recombinant human serine racemase *in vitro* and *ex vivo*.

## Introduction

D-serine (DS), an endogenous D-amino acid, is produced by serine racemase (SR) in glial cells and neurons^1^. SR belongs to the class of pyridoxal-5-phosphate (PLP)-dependent enzymes and it converts the free form of L-serine into DS. The free DS functions as a co-agonist of N-methyl-D aspartate (NMDA) receptor in the brain^1^, retina^2^, keratinocytes^3^ and in chondrocytes^4^. Incorporation of D-serine causes significant changes in the functional properties of many proteins that are detrimental to humans. Racemization of serine residues is known to occur in beta-amyloid senile plaques of Alzheimer’s disease (AD)^5^, human lens protein α- crystallin^6^, human myelin basic protein^7^, osteoarthritic articular and meniscal cartilages^8^, the funnel-web spider toxin^9^, egg ovalbumin^10^ and in skin^3^. The conversion of L-serine to D-serine was conclusively established by the discovery of SR in the rat brain by Wolosker *et al*. in 1999. Active SR is a dimer and needs divalent cations to become functionally active^11^. In addition to serine isomerization, SR also performs α, β-elimination reaction with both L-serine and D-serine to produce pyruvate and ammonia^12^. D-serine is also catabolized by the D-amino acid oxidase (DAAO)^13^. Thus, the D-serine levels are controlled by two enzymes: SR and DAAO.

The NMDA receptors are the major ionotropic glutamate receptors in the central nervous system. They control the flow of Ca^2+^ ions into the cell, which is essential for the synaptic plasticity, synapse formation, rhythmogenesis, and nerve excitation^14^. One of the main features of the NMDA receptor is that its activation requires simultaneous binding of its agonist glutamate and co-agonist glycine. However, DS also binds to NMDA receptor at the glycine site with three-fold higher affinity than glycine^15^. The efficacy of DS to activate the NMDA receptor is 10 times higher in comparison to glycine^16^. Hyper activation of the NMDA receptor takes place in excess of DS^17^. The hyperactivity of the NMDA receptor is involved in many neurological diseases such as Alzheimer’s disease^18,19^ amyotrophic lateral sclerosis^20^, epilepsy^21^, neuropathic pain^22^, and cerebral ischemia^23^.

The NMDA receptor antagonists are often accompanied by adverse effects^24^. Inhibition of SR is another way to regulate the NMDA receptor transmission and is the only way to prevent serine isomerization occurring in proteins in certain pathological conditions including the AD. SR inhibition is proven to be neuro-protective in many situations^25^.

Many studies have used L-*erythro*-3-hydroxyaspartate (L-EHA) and malonic acid as competitive inhibitors of SR^26–29^. However, there is no marketed SR inhibitor available till date. The triterpenoids of *Centella asiatica* (CA) and their derivatives have been shown to exert neuro-protective effects in the experimental model of AD^30–32^. We have used *Centella asiatica* (Gotu kola, an ayurvedic medicinal plant available in India) as the source of the inhibitor of SR.

## Results

### Purification and identification of SR

**Figure 1:**
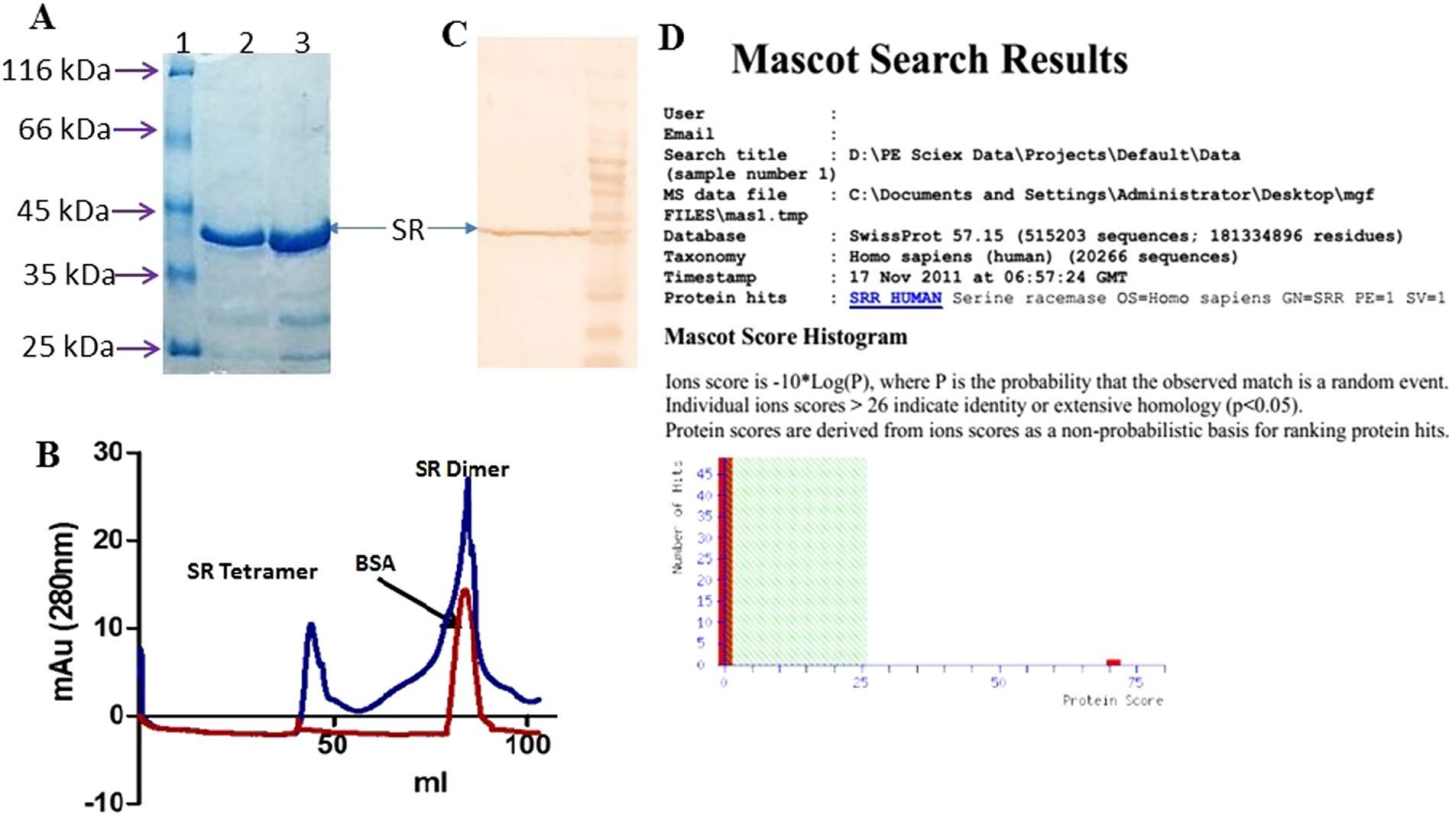
Purification and identification of recombinant SR. (**A**) PAGE analysis of Ni-NTA purified SR (Full size gel is given in Supplementary Fig. S1), (**B**) Size exclusion chromatography of Ni-NTA purified SR showing tetramer and dimer (Full image is given in Supplementary Fig. S1), (**C**) Western blot with anti-His tag antibody (Abcam) and (**D**) Mass spectrometry identification of purified recombinant SR.

37 kDa recombinant human SR was expressed in prokaryotic system and then purified by Ni-NTA affinity and size exclusion chromatography (Fig. 1, panels A and B). The enzyme appeared as a dimer in solution but, in the presence of SDS, it existed as a monomer. The dimer is the active form of the enzyme. The identity of SR was confirmed by western blot analysis and mass spectrometry (Fig. 1, panels C and D).

### CA extract-SR binding analysis using SPR

**Figure 2:**
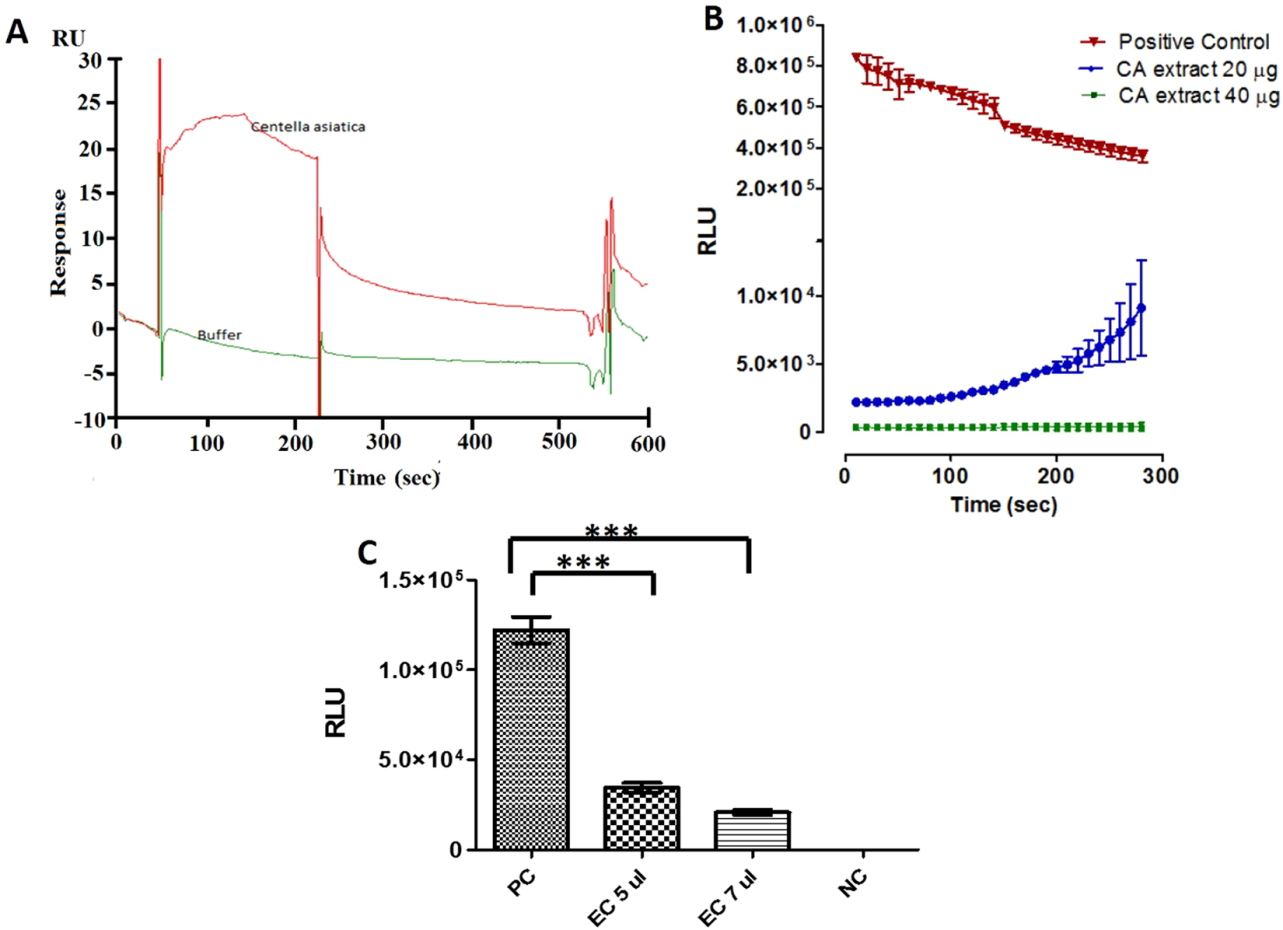
Inhibitory effect of methanol extract of *Centella asiatica* on SR. (**A**) Sensorgram of CA extract binding with immobilized SR, (**B**) Dose-dependent SR inhibition by CA extract. Curves represent positive control (▼), 20 μg/ml (●), 40 μg/ml (■). Data are presented as mean ± SD and (**C**) Inhibitory effect of eluted compound (EC) on SR activity. The assay was carried out with five and seven μl column eluent. Data are presented as mean ± SD.

SPR was performed to screen the plant extracts for direct binding to SR. CA extract showed maximum binding to SR. The sensorgram of SR interaction with CA extract is shown in Fig. 2, panel A. The sensorgram exhibited the compounds in CA extract binding to SR. As the CA extract bound to SR, an increase in response units is seen when compared to buffer.

### Inhibition of SR activity by CA extract

The chemiluminescent assay was used to measure the inhibitory effect of CA extract. SR activity was significantly inhibited in the presence of CA extract. CA extract at a concentration of 20 µg/ml and 40 µg/ml inhibited 85% and 99% of SR activity (p = 0.0001) respectively (Fig. 2, panel B). However, there was a minor rise in the luminescence at 20 µg/ml concentration of CA extract and this could be due to reversible binding of some low affinity inhibitory compounds in CA extract.

### Purification of inhibitors from CA extract

Affinity pull-down was used to purify the inhibitors from the CA extract. His-tagged purified SR protein was immobilized on the Ni-NTA beads. The CA extract was passed through the beads. The bound compounds were eluted using 1 M ammonium bicarbonate. The salt was removed by repeated solvent evaporation leaving salt-free SR-binding compounds.

### Serine racemase inhibition by eluted components from pull down assay

The affinity purified fraction (eluted component, EC) showed significant inhibition of SR (Fig. 2, panel C). The purified column fractions showed dose-dependent response: 5 µl/ml and 7 µl/ml showing 70% and 85% inhibition of SR respectively (p = 0.0001). These fractions were lyophilized and used for identification.

### Identification of purified inhibitors

The eluted compounds were separated on a UHPLC attached to the mass spectrometer. The eluted fraction was run twice (Supplementary Fig. S2). There were essentially four peaks. The first peak at 1.24 mins is the solvent peak. The cluster peaks between 6 and 7 mins did not have any viable MS/MS pattern to match with any database compounds. The peaks at 8.47 and 11.55 mins had MS/MS matches which was used to identify the compounds (Supplementary Fig. S2). The experiment was repeated twice to confirm the peak’s presence and experimental parameters. The compounds had *m/z* values 975.5189 and 505.3539.

### Molecular identification of *m/z* 975.5189

**Figure 3:**
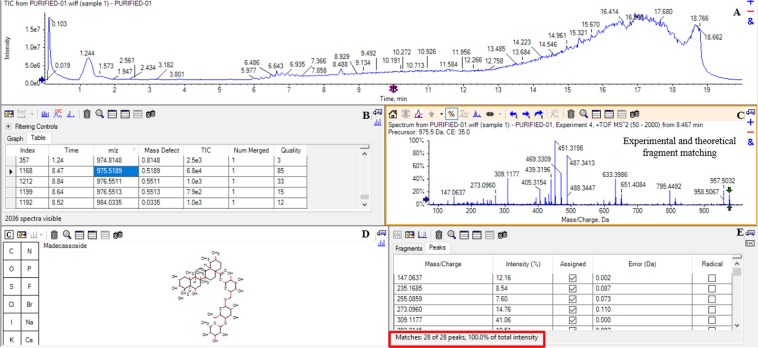
Structural Elucidation of m/z 975.5189 in PeakView Software. (**A**) The elution profile of eluted compound, (**B**) the observed *m/z* and retention time, (**C,D**) the fragmentation pattern and identification of *m/z* 975.5189, (**E**) matched peaks of experimental and theoretical fragments of madecassoside.

The eluted fraction was run in LC-MS-MS (Fig. 3, Panel A). The elution of one compound was noted at 8.47 mins as depicted in panel B. *m/z* value of 975.5189 was obtained for the compound and identification was done by matching the theoretical and experimental fragments of MS/MS pattern. The METLIN software was used to generate a list of all probable compounds using theafore mentioned m/z value. The molfiles of each compound were uploaded in Peakview software and matched to the experimental fragments of m/z 975.51589. The experimental fragmentation pattern is shown in Panel C. A 100% match was found between the madecassoside (METLIN ID 94663) molfile and *m/z* 975.5189 (Panel D and Panel E, Fig. 3) fragmentation pattern. The matched peaks are highlighted in blue color whereas non-matched are highlighted in red color (Panel C, Fig. 3). The *m/z* 975.5189 fragmented to 795.4492, 651.4084, 633.3986, 487.3447 and 451.3198 *m/z*. Other important fragments that were also studied were m/z 633.3986, 488.3447 and 451.3198. They were studied at a retention time of 8.47 mins. *m/z* 633.3986 and 487.3413 also showed a MS/MS spectra fragment with *m/z* 451.3198. The major fragment ions obtained after the loss of the glucose moiety were at *m/z* 795.4492, 651.4084 and 488.3447.

### Molecular identification of *m/z* 505.3539

The second peak was eluted at 11.55 mins corresponding to *m/z* 505.3539 was identified as madecassic acid (Supplementary Fig. S2). Madecassic acid is aglycan part of madecassoside. Major fragments in the spectrum (*m/z* 487, 469 and 451) were investigated, and each *m/z* was also seen in MS mode with the same elution profiles at a retention time of 8.47 mins. MS/MS spectra of *m/z* 487 also produced the fragment *m/z* 451. The *m/z* 505.3539 was identified as compound madecassic acid as described earlier. Since no other peaks in the chromatogram produced viable MS/MS spectra, it was concluded that only two ligands bound to and inhibited SR from CA extract.

### Structural elucidation of madecassoside and madecassic acid by FTIR spectroscopy

**Figure 4:**
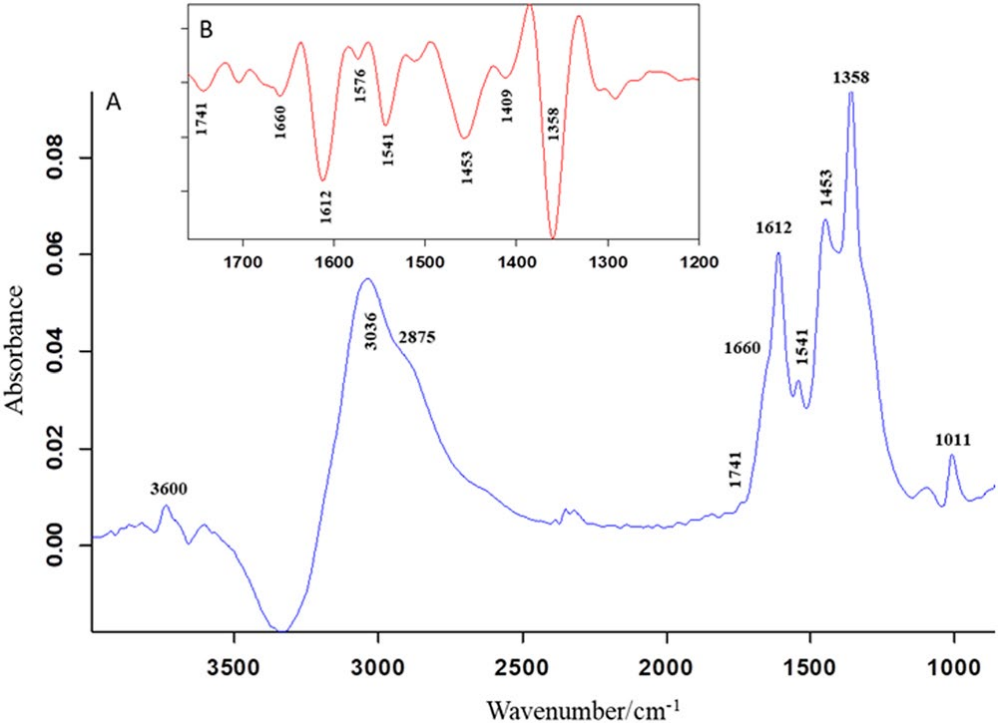
The infrared absorbance (**A**) and respective second derivative (**B**) spectrum of the eluted sample measured at room temperature. The second derivative spectrum was enlarged for better visibility.

Figure 4 shows the absorbance (Panel A) and respective second derivative (Panel B) spectrum of eluted molecules from CA extract. In the absorbance spectrum (4000–900 cm^−1^), we observed 10 major bands. According to the literature^33,34^, we assigned the band at 3600 cm^−1^ to OH stretching vibration, band at 3036 cm^−1^ to =CH vibration from aromatic ring, the broad band near 2875 cm^−1^ to C-H stretching vibration, band at 1741 cm^−1^ to C=O stretching of carbonyl ester, band at 1660 cm^−1^ to C=O stretching vibration of aromatic ring, band at 1612 cm^−1^ to C=C conjugated ring vibration, band at 1541 cm^−1^ to C-N stretching vibration arising may be from imidazole ring, band at 1453 cm^−1^ to C-H wagging vibration, strong band at 1358 cm^−1^ to O-CH wagging or =CH rocking or may be also contributed from cyclic OH vibration, and band at 1011 cm^−1^ to cyclic OH vibration. In the second derivative (Fig. 4B) spectrum (1760–1200 cm^−1^), we have amplified the image for better visibility. We have observed the all bands in this region, discussed above in the absorbance spectrum.

### Post docking madecassoside and madecassic acid with serine racemase

**Figure 5:**
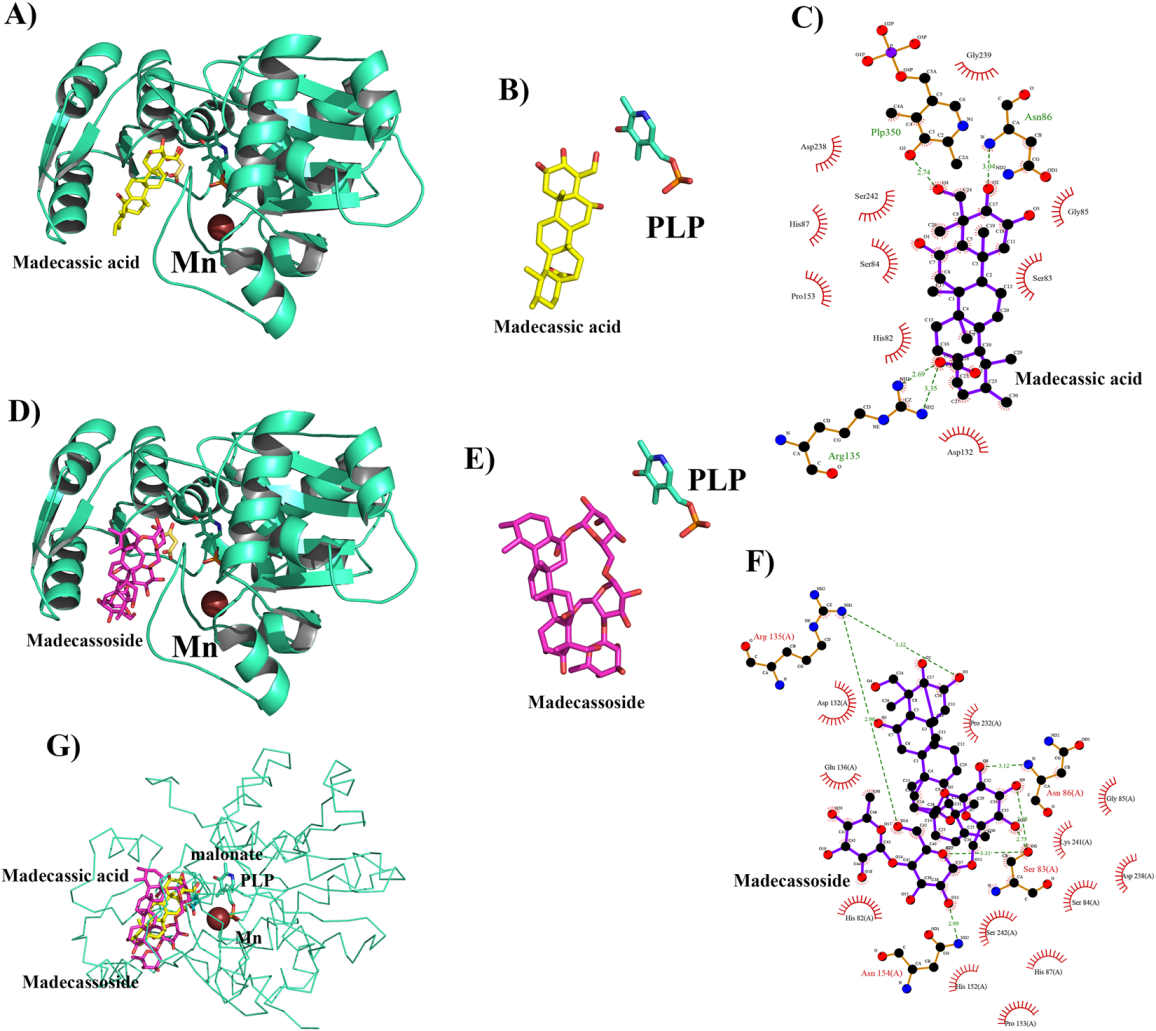
Post-docking interactions between active site residues of serine racemase with madecassic acid and madecassoside. (**A**) Serine racemase is depicted in cartoon view and madecassic acid in stick model in the binding pocket superposed over malonate with manganese and PLP. (**B**) Chemical structure of madecasosside (**C**) Madecassic acid interaction with active site residues of serine racemase where hydrogen bonds are indicated by dashed lines between the atoms involved, while hydrophobic contacts are represented by an arc with spokes radiating towards the ligand atoms they contact. (**D–F**) shows the interactions of madecassoside with SR. (**G**) shows the binding pose of both the ligands in the active site of SR.

The docking results displayed binding between madecassoside and serine racemase. The Glide score of the best scoring conformer was −12.58 kcal/mol and X-score was −10.30 kcal/mol (Supplementary Table S1). Further structural analysis presented 6 hydrogen bond interactions and 12 hydrophobic interactions between the madecassoside and SR (Fig. 5F). The docking simulations suggested that the binding of madecassoside is mostly driven by hydrogen bond network which helps it bind better as shown in Fig. 5D,F. Madecassoside establishes two hydrogen bonds with the hydroxyl group of Ser- 83 and makes one hydrogen bond with the amino group of Asn-86. It also participates with Asn-154 via carboxamide side chain. The guanidinium group of Arg-135 also makes two hydrogen bonds with the compound, one with the aglycan moiety, and one with the sugar moiety. Amino acid Ser 83 is present in/at the substrate binding loop of the enzyme. In the closed conformation, it mainly acts as a recognition site for the ligand of the enzyme. This is a constitutional feature shared by various type II PLP-dependent enzymes^35^.

The docking of ligand, madecassic acid showed good binding with SR. The Glide score and the X-score for madecassic acid were −6.48 kcal/mol and −8.56 kcal/mol respectively (Supplementary Table S1). There were 3 hydrogen bond interactions and 9 hydrophobic interactions between madecassic acid and SR. Madecassic acid interacts with Asn-86 via a carboxylic group, and it also interacts with PLP at the bottom of the pocket. Compared with the binding pose of madecassoside, madecassic acid shows loss of direct hydrogen bond interactions with Ser-83 and Asn-154. Interestingly, the binding pose of madecassic acid and madecassoside are similar to the binding pose of co-crystallized malonate (Fig. 5, Panels A, D and G). The orientations of madecassoside and madecassic acid with respect to PLP when complexed with SR are given in Fig. 5 (panels B and E). The interactions of these two ligands are shown in Panels C and F of Fig. 5. The Glide score and X-score for malonate were −5.96 kcal/mol and −6.12 kcal/mol respectively and are less than those for madecassoside and madecassic acid (Supplementary Table S1).

### Molecular dynamic simulation of the protein-ligand complexes

**Figure 6:**
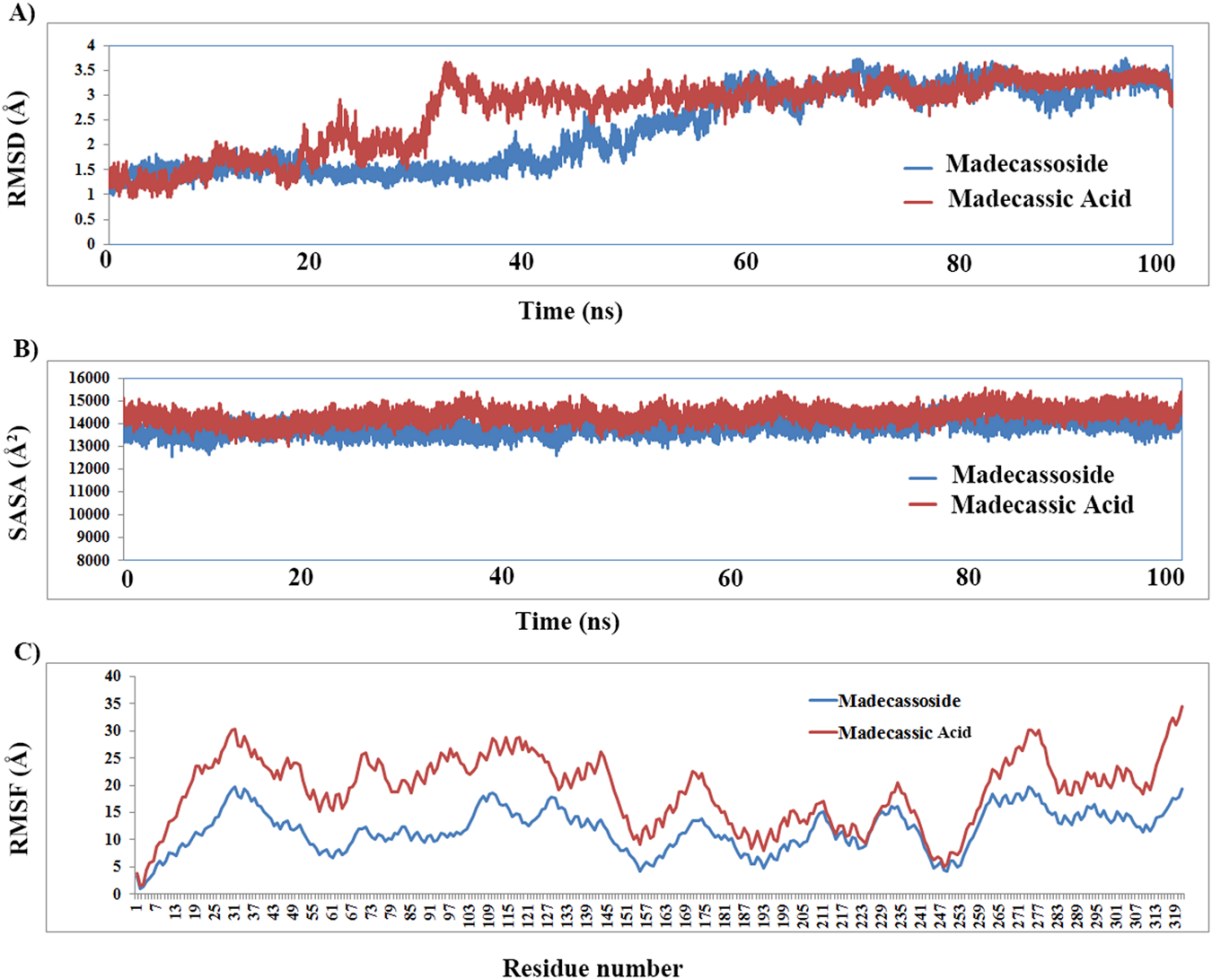
The molecular dynamics simulations of serine racemase with madecassic acid and madecassoside over a time scale of 100 ns. (**A**) Root mean square deviations of protein- ligand complexes from the initial starting structure. (**B**) Shows the solvent accessibility surface area of both the systems. (**C**) Shows the root mean square fluctuations of all the residues of SR in the presence of madecassic acid and madecassoside.

The docking poses showed proper binding of both the ligands at the active site of serine racemase. The complexes were further subjected to molecular dynamics simulations to understand the behavior of the ligand molecule in motion mimicking physiological conditions for a time scale of 100 ns. The ligand-protein complexes of SR merged in a water box with 51389 (madecassic acid) and 50677 (madecassoside) atoms respectively showed stability after 40 ns of production dynamics (Fig. 6). Simulation of madecassic acid showed slightly higher overall fluctuations compared to madecassoside. Both the complexes stabilized at a rmsd around 3.5 Å (Fig. 6A) deviated from the starting structure. We also calculated the solvent accessibility surface area (SASA) for both the systems over the period of 100 ns. The simulated structure of madecassic acid showed average SASA of 14355.69 Å compared to madecassoside which showed an average of 13830.73 Å. The SASA calculations clearly indicated (Fig. 5B) madecassoside binds with serine racemase and retains most of the binding space available compared to madecassic acid. The root mean square fluctuations suggested that fluctuations of the residues of serine racemase are similar in both the systems. The madecassic acid complex shows higher fluctuations especially in the N-terminal region (Fig. 5C). The binding analysis of the complexed structure through molecular dynamics simulations suggested that the binding of madecassoside is mostly driven by electrostatic energies. The trajectories of both the molecules stabilize at a rmsd of 3.5 Å and have shown similar conformation. The conformational changes brought by the binding of madecassoside caused the structural changes of 2 Å around 35 ns timescale compared to 60 ns in the case of madecassic acid. The stabilization of both the systems suggests that the binding of madecassoside to SR is more stable in the overall timescale of the simulations.

Using the molecular dynamics trajectories of both madecassic acid and madecassoside, the binding free energy calculations were carried out by molecular mechanics, the generalized born model commonly known as MM/GBSA. Madecassoside presented better binding free energy compared to madecassic acid (Supplementary Table S2) which was evident from the analysis of MD simulation data. The detailed binding free energy analysis of both the molecules and the individual contribution of residues are shown in the Supplementary Data (Supplementary Table S3).

### *In vitro* inhibition of SR by madecassoside

The *in-silico* study revealed that madecassoside has the higher binding affinity towards SR than madecassic acid. We further attempted to study the inhibitory effect of madecassoside on SR by calculating the IC_50_ value of madecassoside with *in vitro* chemiluminscent based SR activity assay.

**Figure 7:**
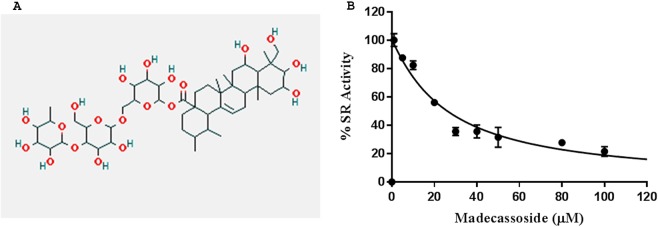
(**A**) Chemical structure of identified SR inhibitor madecassoside. (**B**) The graph showing the IC_50_ curve of madecassoside for SR inhibition with an IC50 value 26 µM.

Madecassoside inhibited recombinant human SR at all the concentrations taken in the experiment. The positive and negative controls worked efficiently, positive control showing 10 fold higher luminescence than the highest luminescence seen in experimental reactions and the negative control showing luminescence lower than the lowest luminescence seen in experimental reactions. A concentration dependent inhibition was observed. The IC_50_ value for madecassoside is 26 µM (Fig. 7B). The IC50 value for malonate, which is a well-known inhibitor of serine racemase, is 449 µM (Supplementary Fig S6). This demonstrates madecassoside is a far better inhibitor of SR in comparison to malonate. Also, inhibition of SR by MDS is specific as other PLP dependent enzymes serine dehydratase and aspartate transcarbamylase are not inhibited by MDS. (Supplementary Fig S7).

### *E*x *vivo* inhibition of SR by madecassoside

**Figure 8:**
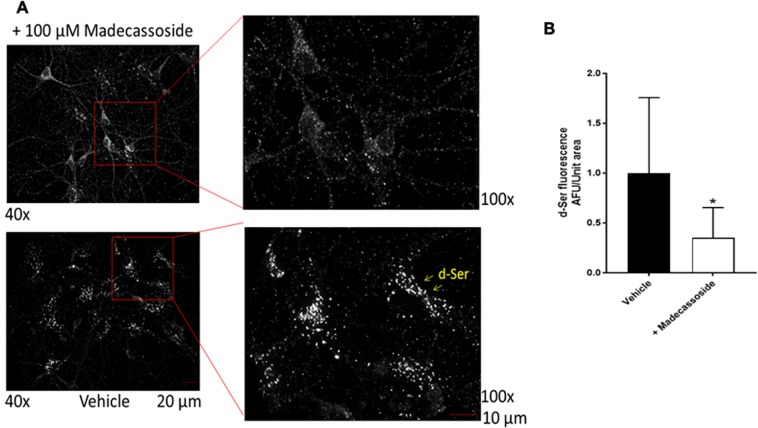
*Ex viv*o Inhibition of serine racemase. (**A**) Cultured hippocampal neurons were treated with vehicle or madecassoside and stained with D-serine antibody to visualize D-serine production. Note the D-serine puncta in the neuronal soma and the processes in the vehicle-treated neurons (bottom). However, the total number of puncta significantly disappeared upon madecassoside treatment (top) indicating an inhibition of SR. Two independent cultures were used. (**B**) Quantification showing significant reduction of D-serine fluorescence in madecassoside treated cell compared to control cells.

To further validate the *in vitro* results in an *ex vivo* setting, we tested the inhibitory effect of madecassoside in murine cultured hippocampal neurons. We used a culture protocol that enriches neurons over other non-neuronal cells^36^. We treated the cultured neurons with either the vehicle or madecassoside followed by immunostaining with D- serine specific antibody and visualized under the fluorescence microscope. Endogenous D- serine appeared as puncta in the soma as well as in the neuronal processes of the vehicle- treated neurons (Fig. 8A) indicating the presence of a functionally active SR inside the cells. The punctate distribution of D-serine in neurons has been reported earlier^37^. However, in the madecassoside treated neurons, the D-serine puncta have disappeared or significantly reduced from the neuronal soma as well as the neurites suggesting a depletion of D-serine inside the neurons (Fig. 8B). As the production of D-serine in the cells is solely dependent on the conversion of L-serine by the functional SR, our *ex vivo* experiments suggest inhibition of SR by madecassoside in the treated neurons.

## Discussion

Here we report an innovative method of purifying ligands from plants and identifying them using mass spectrometry. This new approach has been applied to screen, purify inhibitor and identify the inhibitor from CA plant extract. Our method enables to purify and identify the potential ligand for disease target in about two-week experimental time. This shortened experimental time in identifying the ligand for a disease target is a clear advantage over the conventional approaches, such as Bioassay-Guided Fractionation^38,39^. Our method has added advantages too. The identification of active extract can be automated to be used in a high throughput format. The targets can be multiplexed so that the same extract is screened for ligands for many targets simultaneously. The ligands can be purified using affinity methods. The use of mass spectrometry quickens the process of identifying the ligand. This study also validates the medicinal plants for a molecular target. *In silico* protein and small molecule interaction, *in vitro* and *ex vivo* experiment were used to validate the inhibitory activity of purified compounds.

D-serine is an important physiological and pathological neurotransmitter^40^. Serine racemase is the only biosynthetic source of neurotransmitter D-serine in the mammalian brain. Its involvement in the over-activation of NMDA receptor is unequivocally proved. The NMDA receptor mediated excitotoxicity is involved in a variety of severe and chronic neurodegenerative diseases. Inhibition of serine racemase could be an alternative way to regulate NMDA receptor transmission. Some intrinsic mechanisms are in place to control the DS level such as translocation of SR to the plasma membrane^41^, nuclear compartmentalization of SR^42^, and catabolism by D-amino acid oxidase^13^. It has been established that reduction of DS level is useful in ischemia^43^, neurotoxicity^19^, seizures^44^, neuropathic pain^45^, excitotoxic retinal damage^46^, and other neurological disorders. CA has been shown to have a positive effect in all the above situations^47–50^.

The affinity chromatography method separated the ligands binding to serine racemase. We identified these compounds using mass spectrometry. The complexities of the mass spectrometric methods and tools can at times lead to some uncertainties in MS results. Hence, we have used FTIR characterization to validate the LC-MS dentification of the compounds present in eluted fractions from affinity chromatography. The band at 1741 cm^−1^ in the second derivative of FTIR spectrum is assigned to carbonyl ester arise from madecassoside whereas, the bands at 1576 cm^−1^ and 1409 cm^−1^ are assigned to asymmetric and symmetric COO- vibration respectively from the madecassic acid. The observed absorption of carbonyl ester was higher than the acid indicating higher amount of madecassoside than madecassic acid. These infrared findings support the results obtained from mass spectrometry: the eluted sample contained two molecules, majorly madecassoside and madecassic acid in lesser amount. Madecassoside and madecassic acid are the two compounds identified from the CA plant extract binding to SR. Both the compounds are the triterpenoids of plant CA^51^.

Madecassoside is a pentacyclic triterpenoid saponin, polar in nature and freely soluble in water (Fig. 7A). Madecassoside upon hydrolysis yields a glycan part and madecassic acid. To compare the binding affinity, *in silico* binding studies were performed with madecassoside, madecassic acid along with the previously reported competitive inhibitor malonate. One of the limitations of the docking simulations is that it doesn’t take account of the conformational flexibility of the enzyme present during binding. Hence, we have used the closed conformation of SR in our docking studies and MD calculations. The docking studies confirmed that all three compounds - malonate, madecassoside and madecassic acid are capable of binding to the active site of SR and the simulations over 100 ns on each system suggested that the they are stable inside the pocket. All of them bind with strong binding energy (binding free energy). The post simulation comparative analysis of high throughput structural data of madecassoside and madecassic acid suggested that madecassoside occupies more SASA active and forms stable interactions with SR. In comparison to malonate, madecassoside and madecassic acid form a greater number of hydrogen bonds and hydrophobic interactions with the active site of SR. In the case of madecassoside, G-score and X-score are very high, indicating the madecassoside bind to the protein active site with higher affinity in comparison to malonate (Supplementary Table S1). The fused ring structure of madecassoside stacks into the active site of SR protein and makes hydrogen bonds. The sugar moieties present in madecassoside augment the interactions with the protein active site. The madecassic acid interacts with SR with lesser affinity when compared to madecassoside, possibly due to lack of glycan part, it is unable to make additional interaction with the polar residues of the active site. The present work concerns with the rapid identification of SR inhibitors present in the medicinal plant extracts and not designing an inhibitor. Hence, we did not consider the allosteric modulators on substrate site binding of SR.

In order to check the specificity of madecassoside to SR, we conducted molecular docking studies of madecassoside and two PLP dependent enzymes other than SR: serine dehydratase (1P5J) and aspartate racemase (5HRC). Molecular docking of madecassoside with both serine dehydratase and aspartate racemase showed significantly low binding score when compared to SR. Thus, madecassoside is a highly specific inhibitor of SR (Supplementary Fig. S6 and Supplementary Table S5). This is confirmed by experimental results (Fig. 7B, Spplementary Fig. S7). The enzyme inhibition assay exhibits that madecassoside is an inhibitor of the human SR. The IC50 value for madecassoside is 26 µM, which is the lowest IC50 among SR inhibitors reported so far. This establishes the potency of madecassoside to serve as a strong competitive inhibitor of SR. Several research groups such as Vorlova & coworkers, Mori & coworkers, Beato & coworkers, Hoffman & coworkers^28,52–54^ have made attempts to discover and design inhibitors for serine racemase. The least IC50 value reported for any inhibitor of SR by far is 57 µM^52^ (Supplementary Table 4). Hence, we are reporting the most potent inhibitor of SR known till date.

Madecassoside has already been reported to have the neuro-protective effect. In neuronal cells, madecassoside significantly decreases the level of D-serine. It reduces the Aβ induced inflammatory response^55^. The anti-inflammatory activity of madecassoside may be responsible for reducing ischemia-reperfusion injury and useful in reducing the damage caused by stroke^56^. In the animal model of ALS, madecassoside has shown anti-apoptotic and anti-oxidative effects^57^. However, the mechanism behind these neuroprotective effects of madecassoside has not been reported till date. In our opinion, the neuroprotective effect of madecassoside is associated with SR inhibition. The finding of our work, inhibition of SR by madecassoside explains the mechanism of the neuroprotective effect of the same.

Lin *et al*., have shown that administration of madecassoside markedly decrease the level of NO and nNOS in the D-gal treated mice. However, the underlying mechanism is not yet clear^58^. It has been reported that nNOS is activated by increased Ca^2+^ mediated by NMDA receptor activation in neuronal cells^59,60^. The NMDA receptor subunit NR2B by its interaction with nNOSα acts on the postsynaptic density protein-95 (PSD-95) with the help of specific PDZ domains. This interaction has been considered a direct link between the action of NMDA receptors on NO production^61,62^. By these findings, we conclude that madecassoside is not directly involved in the reduction of NO level in the cell. However, this effect of madecassoside could be mediated by SR inhibition. The reduction of SR activity and D-serine level regulate the activation of NMDA receptor mediated diseases^63^.

In our experiments as well as the previous experiments involving neuronal cells, the exogenously given madecassoside is taken up by the neurons. The animal experiments establish that this inhibitor can cross the blood-brain barrier. Thus, the oral ingestion of madecassoside can reduce the production of neuronal D-serine. Although the usage of glutaraldehyde crosslinked antibody has some reservation in case of solid tissues, the cultured-neurons in our experiments have displayed a similar punctate distribution of D-serine in neurons that have been reported earlier^64^.

High-end technologies are used by which we are able to identify the active compound of a natural extract (screen), obtain the active component and confirm it – all in a matter of one week experimental time. The conventional procedure could have taken at least six months to complete. That is one clear advantage over the conventional approaches. The method described in our work is a significant step forward in the drug discovery pipeline. Fifty percent of molecular medicines are direct plant molecules. Hence, this approach could be used to screen the plant extracts for multiple targets. *Ex vivo* or animal experiments can be used to validate the ligands.

## Methods

### Serine racemase expression and purification

Human SR gene (Accession: NM_021947.1) was commercially purchased from Gene Copoeia, USA. The gene (1020 bp) was PCR amplified using gene specific primers and subcloned into pProEX HTa vector. The protein was expressed in *E. coli* rosetta cells. The protein was purified using Ni-NTA affinity chromatography in 50 mM Tris-HCl, pH 8.0 containing 25 µM PLP (with 300 mM imidazole as eluting agent). It was further purified using Sephadex S-200 (GE Healthcare, USA) in AKTA Prime (GE Healthcare, USA) with a flow rate of 1 ml/min of 20 mM Tris-HCl, 150 mM NaCl, and 15 μM PLP pH 8.0. The purified protein was identified by mass spectrometry and was resolved in SDS-PAGE to test.

### Sample collection and methanolic plant extract preparation

Medicinal plants were collected from Mother India Nursery, Najafgarh, New Delhi. The plants were identified by Dr. Vandana Mishra, Department of Environmental Studies, University of Delhi. 10 g of shadow-dried plants were extracted with 250 ml of methanol for 16 hrs with constant agitation at room temperature. The residues were re-extracted with 150 ml of methanol. The supernatants were pooled and evaporated to dryness. The dried samples were weighed and stored at −20 °C in a desiccator.

### SPR based screening of plant extracts

The experiments were performed on a BIAcore T100 at the John F. Welch Technology Centre, Bangalore, India. The recombinant SR (100 μg/ml) was immobilized on a CM5 chip (GE Healthcare, USA) using the amine coupling method according to the manufacturer’s protocol. The sample extracts were dissolved in PBS at a concentration of 1 mg/ml. The sample tray was maintained at 4 °C while the binding analysis was performed at 25 °C.

### SR enzyme activity assay

The chemiluminescent assay for SR was carried out using previously reported method by Wolosker *et al*.^1^. SR activity assay reaction was carried out in 50 mMTris-HCl, pH 8.0 buffer containing 20 µg of SR protein, 200 µm pyridoxal 50 -phosphate, 1 mM EDTA, 2 mM DL-dithiothreitol, 5 mM L-serine and different concentrations of inhibitor (5 µM to 100 µM). Heat-inactivated SR was used as negative control in the assay. All reactions were kept in duplicates. The reaction mixtures were incubated at 37 °C for 3 hours. The reaction is stopped by heating the reaction mixtures at 95 °C for 10 minutes and then centrifuged at 13,000 rpm for 10 minutes. A reaction mixture, with final volume of 100 µl was made with 30 µl sample supernatant, 16 µM luminol, 8 units of HRP, 8 µl of DAAO (15 U/0.1 ml) and 50 mM Tris buffer, pH 8.8. The chemilumniescence was detected using luminometer (Spectramax i3). Since SR inhibition assays were performed using plant extract, eluted fraction, and purified compound madecassoside (sigma-M6949), their concentrations used during the experiments are mentioned below:

For SR inhibition assay using plant extract, 20 µg and 40 µg of Centella asiatica extract was used. For SR inhibition assay using eluted fractioned, 5 µl and 7 µl of eluted fraction from pull down assay was used. For SR inhibition assay using purified compound madecassoside (MDS), 5, 10, 20, 30, 40, 50, 80, 100 µM of MDS was used. IC50 value for madecassoside was calculated using GraphPad Prism 7. The percentage inhibition for each concentration of *Centella asiatica* (CA) extract and eluted fraction was calculated using the following formula:

$$ \% \,{\rm{inhibition}}=[({\rm{normal}}\,{\rm{activity}}-{\rm{inhibited}}\,{\rm{activity}})/({\rm{normal}}\,{\rm{activity}})]\times 100 \% $$

### Pull-down experiment

The dried methanol extract of CA was dissolved at a concentration of 10 mg/ml in sodium phosphate buffer containing 150 mM NaCl. The purified recombinant SR (5 mg) was immobilized on Ni-NTA-agarose (2 ml of 50% slurry). The extract, 10 mg/ml PB (10 mM, pH 7.4), were passed through the column. After washing the unbound compounds, the bound compound was eluted with 1 M (NH_4_)_2_CO_3_ and lyophilized. The purified compounds were identified by mass spectrometric methods and validated for activity.

### Mass spectrometric identification of inhibitor from CA plant

The crude extract of the plant and purified compounds eluted from the pull-down experiment was analyzed by mass spectrometry^65^. The crude extract and the eluted compounds were re-suspended in 0.1% formic acid solution and injected into a Shimadzu Prominence SIL HTC system equipped with a C18 column, 1.8 µm, 2.1 × 100 mm (ACQUITY UPLC HSS T3 Column).

A gradient is run from 100% water to 100% acetonitrile, with 0.1% formic acid was used at a flow rate of 0.3 ml/min over 15 mins. Injection volume was 5 μl. A completely generic method was used for data acquisition on all compounds and all samples. A Triple TOF 5600 system with a DuoSpray Source (electrospray ionization) was used for data acquisition in positive mode, over a mass range of 100–1000 *m/z*. A TOF MS survey scan experiment (100 ms) incorporating IDA set to monitor was performed with a collision energy of 45 eV and a spread of ±15 eV.

Data was processed using PeakView Software for features identification and ID confirmation. The MS and MS/MS data were further evaluated using structural elucidation tool within PeakView Software. The confirmation of compound was done based on structure elucidation by theoretical and experimental fragment matching. Accuracy of mass data is generally <2 ppm. The percentage matching was obtained, and a list of all the fragments assigned by matching theoretical fragments of the structure to the experimental spectrum was displayed.

### FTIR spectroscopy

FTIR spectroscopy was used to identify the eluted compounds from the SR affinity column. FTIR spectra were recorded at 4 cm^−1^ resolution on a Bruker Vertex 27 FTIR spectrometer combined with attenuated total reflection elements (ATR) and equipped with a DTGS detector. The IR instrument was purged with N_2_ gas. The eluted sample was placed on a diamond ATR reflection element, and the sample spectrum (64 scans) was ratioed against a 128 scan background spectrum. The experiment was performed three times in the liquid sample at room temperature using the spectrometer software OPUS (Bruker, Germany). All the spectral data were collected in the 4000–900 cm^−1^ region.

### Molecular docking and molecular dynamics simulations

A structural comparison of SR bound to malonate with a SR in the free form (PDB code: 5X2L)^66^ clearly suggests that the enzyme undergoes an open-closed conformational change when a ligand is present at the active site. The structure of SR in an open conformation (PDB code: 3HMK) changes to a closed conformation upon formation of a complex with the competitive inhibitor malonate (PDB code: 3L6C). This is one of the known closed confirmation of SR in presence of the inhibitor. The malonate complex crystal structure of the Human SR was retrieved from Protein Data Bank ID: 3L6C. The dimeric molecules are related by C2 symmetry in the unit cell. The two molecules are structurally equivalent. Hence, the monomeric molecule is considered for the calculations. The malonate molecules were removed from the complex. The structure was further optimized by energy minimization in Schrodinger Glide by using OPLS2005 as a force field. The docking grid of 12 Å was defined using active site residues in a cubic space of around the ligand which is interacted with malonate. The residues selected for grid generation in the protein structure are R-135, S-242, S-84, S- 83, H-87, D-132, E-136, P-231, K-241, H-82, P-153, D-238, G-239, N-86, N-154, G-85, N-229, S-243, H-152 and PLP-LYS-56. Ligand interactions were calculated using Ligplot, and binding affinities were calculated using the program X-score (kcal/mol). MD simulations were also done to validate the docking simulations and to identify the crucial binding residues in the receptor for the binding of ligand. To further perform classical molecular dynamics simulations, docking poses which showed maximum glide score in docking simulations (SR-Compound Complexes) were used. AMBER suite antechamber module was used to created parameters for both the compounds. The partial atomic charges of the ligand were generated using the restrained electrostatic potential (RESP) and the drug molecule’s parameters by AMBER force field (GAFF)^67^. The protein was described and then neutralized by addition of hydrogen atoms and counter ions, using standard AMBER force field for bioorganic systems (ff12B). Xelap was used to make input files for dynamics, energy minimization and analysis. Atomistic TIP3P water box with edges 12 Å from the complex were used for solvation of both the systems. AMBER molecular dynamics suite version was used for all the simulations^68^. A detailed description of Molecular Docking Simulations, Molecular Dynamics Simulations, free energy calculations and per residue interaction decomposition are shown in Supplementary Data.

### *Ex-vivo* neuronal SR inhibition

Primary hippocampal neurons were cultured from CD1 mice pups as described by Kaech and Banker^36^. All animal studies were performed in accordance with University of California guidelines, under a protocol approved by UCSD IACUC (Institutional Animal Care and Use Committee under protocol S08168). Briefly, hippocampi, dissected out from P0-P1 mice were gently dissociated in 0.25% Trypsin- EDTA at 37 °C for 15 mins. After 15 mins, enzymatic digestion was blocked with 30% FBS prepared in PBS. Dissociated neurons were plated on poly-D-lysine coated glass- bottom dishes (MatTek) at a density of 25,000 cells/cm^2^, and maintained in Neurobasal/B27 medium with 5% CO_2_ and 80% humidity.

DIV3 neurons were treated every 24 hrs with freshly prepared 100 μM madecassoside in the base medium for four days. On DIV7 both the drug-treated and the vehicle-treated neurons were fixed in 4% PFA for 10 mins. After extracting with 0.2% Triton-X-100 for 10 mins, neurons were blocked in 5% FBS for 2 hrs at RT. Neurons were incubated with the D-Serine antibody (1:200 dilution, AB5917, Millipore) for 2 hrs at RT. After three washes with PBS, cells were blocked with blocking solution for 30 mins and incubated with anti-rabbit secondary antibody tagged to Alexa-594 (1:1000, Molecular Probes) for 1 hr at RT. Neurons were washed in PBS and mounted with a mounting solution.

Multiple Z-stack images were acquired using an Olympus IX81 inverted epifluorescence microscope with 40x and 100x objectives (imaging parameters: 0.2 μm z-step, 0.5 ms exposure and 1 × 1 binning). Images were deconvolved, subjected to a maximum intensity projection and processed using Metamorph (Molecular Devices USA) and ImageJ software (https://imagej.nih.gov/ij). Integrated intensities of D-serine puncta were calculated and compared the values between the drug-treated to the vehicle-treated neurons.

## Supplementary information

Supplementary File.
