# Supplementary material for: Accelerated identification of serine racemase inhibitor from Centella asiatica
Source: Sci Rep. 2020 Mar 13;10:4640. doi: 10.1038/s41598-020-61494-1 (PMC7070078; doi:10.1038/s41598-020-61494-1)
Supplement: Supplementary file 1 — Supplementary File. [file 41598_2020_61494_MOESM1_ESM.docx]

Accelerated identification of serine racemase inhibitor from *Centella asiatica*

Komal Rani^a^, Mitali Tyagi^a^, Mohit Mazumder^b^, Akanksha Singh^c^, Annaian Shanmugam^d^, Krishna Dalal^e$^, Manoj Pillai^c^, Gourinath Samudrala^b^, Saroj Kumar^a^, Alagiri Srinivasan^a*#^

1. Department of Biophysics, All India Institute of Medical Sciences, New Delhi, India
2. School of Life Sciences, Jawaharlal Nehru University, New Delhi, India
3. Sciex, Gurgaon, India
4. Centre of Advanced Study in Marine Biology, Faculty of Marine Sciences, Annamalai University, Parangipettai, India
5. All India Institute of Ayurveda, New Delhi, India

* Author to whom correspondence should be addressed

# Present address: Department of Biochemistry, Jamia Hamdard, Hamdard Nagar, New Delhi, India

$ Deceased

B

A

1 2

**Figure S1: A depicts the full length SDS PAGE of serine racemase. Lane 1 is ladder while lanes 2 and 3 are Purified SR after protein purification. B depicts the western blot of serine racemase. Lane 1 is SR protein and lane 2 is ladder.**

116 kDa

35 kDa

25 kDa

1 2 3


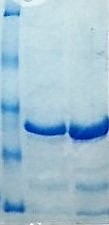

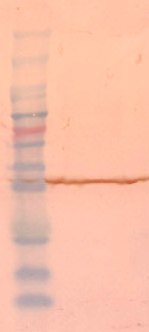


66 kDa

45 kDa


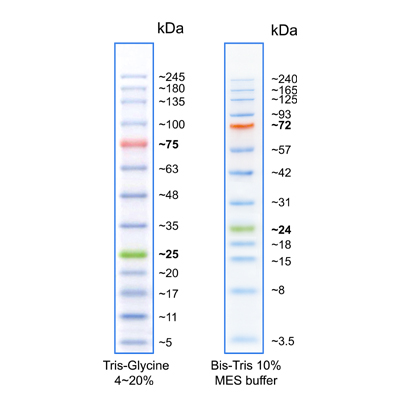


SR


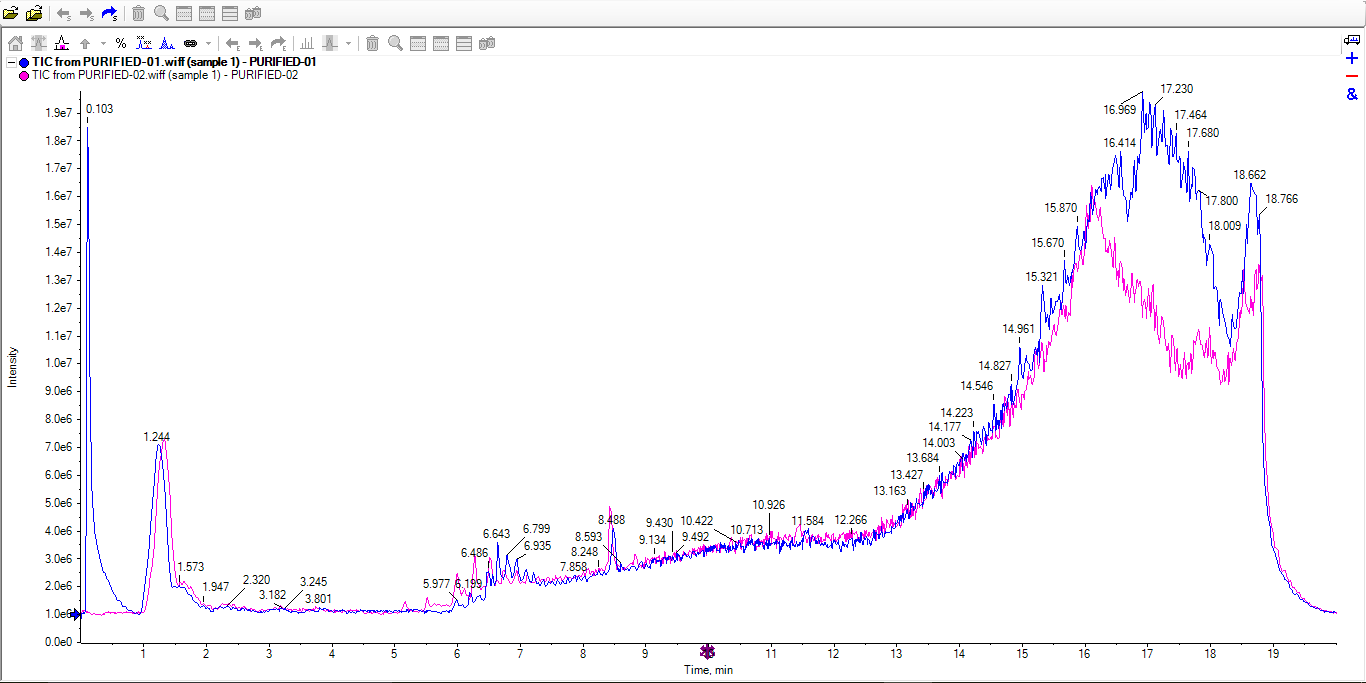


**Figure S2: Chromatogram of isolated compound from *Centella asiatica* after pull down assay. Duplicate runs are shown in blue and pink.**

**
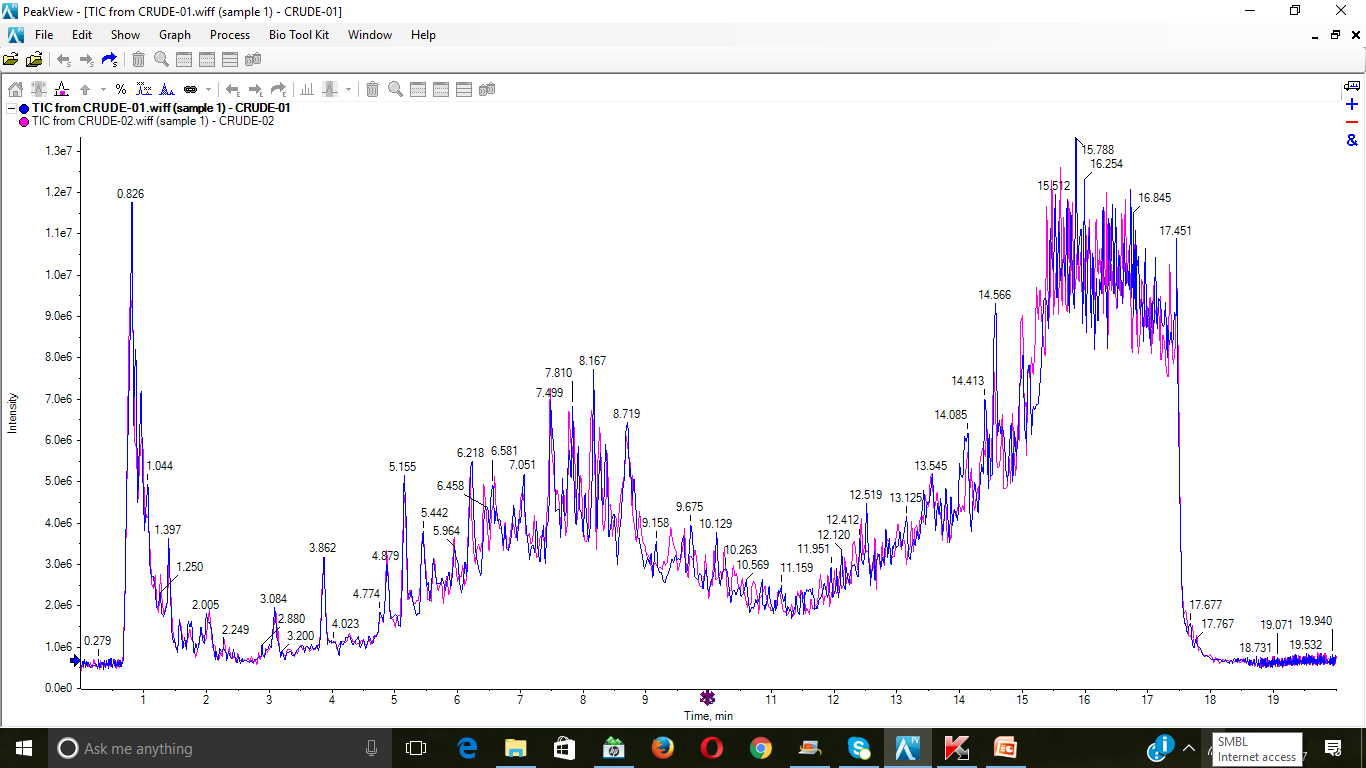
Figure S3: Chromatogram of crude extract of *Centella asiatica*. Duplicate runs of crude extract are shown in blue and pink.**


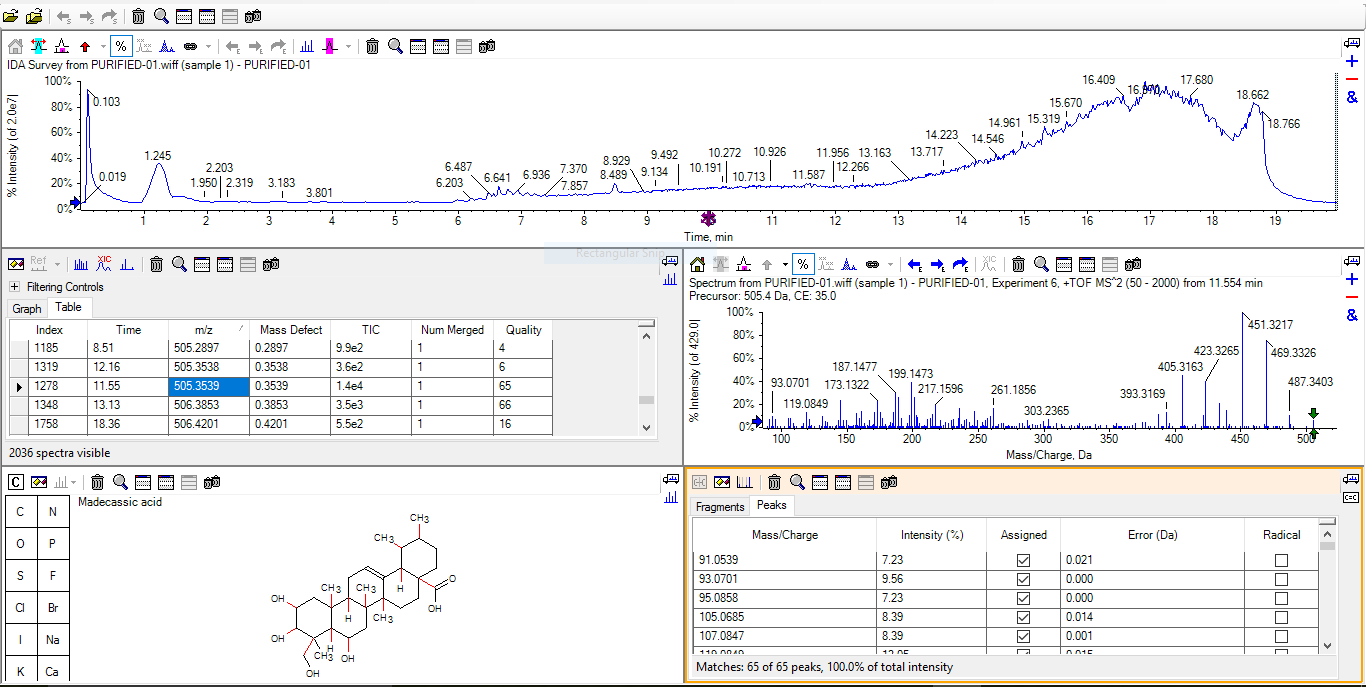


Experimental and theoretical fragment matching

**A**

**B**

**C**

**D**

**E**

**Figure S4: Structural Elucidation of m/z 505.3539 in Peak View Software. The panel (A) indicates the elution pattern, panel (B) indicates the *m/z* and retention time, panel (C) indicates the fragmentation pattern, panel (D) indicates the identity, and panel (E) shows peak matching of both experimental and theoretical fragments for the compound are shown, madecassic acid.**

**Supplementary Methods**

**Molecular docking**

The malonate complex crystal structure of the mammalian serine racemase was retrieved from Protein data bank ID: 3L6C and the complex were removed. The structure was further optimized by energy minimization in Schrodinger Glide by using OPLS2005 as force field. The docking grid of 12 Å was defined using active site residues in a cubic space of around the ligand which are interacted with malonate. The residues selected for grid generation in the protein structure are R-135, S-242, S-84, S-83, H-87, D-132, E-136, P-231, K-241, H-82, P-153, D-238, G-239, N-86, N-154, G-85, N-229, S-243, H-152 and PLP-LYS-56. The protein-ligand docking was performed with two small compound molecules obtained from Pubchem database [(https://pubchem.ncbi.nlm.nih.gov).](file:///G:\(https:\pubchem.ncbi.nlm.nih.gov\)) Energy minimization and dynamic simulations were performed to optimize the docking. For accurate calculations, we have used the extra precision module (XP) for glide docking. Ligand interactions were calculated using Ligplot and binding affinity was calculated using the program X-score (kcal/mol). Solvent accessible surface area was checked using Pdbsum server.

**Molecular dynamics simulations**

MD simulations were also done to validate the docking simulations and to identify the crucial binding residues in the receptor for the binding of ligand. The poses with highest glide scores obtained from the docking simulations (serine racemase-drug complexes) were further subjected to classical molecular dynamics simulations. Parameters for both the drug molecules (in complex with serine racemase) were generated using antechamber module of AMBER suite. The restrained electrostatic potential (RESP) was used to describe the partial atomic charges of the ligands. The general AMBER force field (GAFF) was used to describe the parameters of drug molecules. The standard AMBER force field for bioorganic systems (ff12B) was employed to describe the protein, followed by the addition of hydrogen atoms and counter ions to neutralize the system. The input files for energy minimization, dynamics and analysis were prepared with xleap. Both systems were solvated using atomistic TIP3P water in a box with edges at least 12 Å from the complex. All simulations were performed using AMBER molecular dynamics suite version.

For the equilibration and subsequent production runs, the SHAKE algorithm was employed on all atoms covalently bonded to a hydrogen atom, allowing for an integration time step of 2 fs. The system was gently annealed from 0 K to 300 K over a period of 50 ps using a Langevin thermostat with a coupling coefficient of 1.0ps and 50ps of density equilibration with weak restraints. The system was again equilibrated for 5ns without any restrains. The production phase of the simulations was run without any restraints for a total of 100 ns on each system. Coordinates and energy values were collected every 10 ps throughout the simulations.

**Binding free energy calculations**

The binding free energies of serine racemase for both the drug molecules were analyzed by the MM/GB SA scripts, integrated in the AMBER 12 software package. In this procedure, snapshots were first extracted from the obtained trajectories. For each snapshot, free energy is calculated for the protein, ligand, and complex using single trajectory approach. The binding free energy was computed as the difference:

ΔG_bind_ = G_complex_ − G_protein_− G_ligand_

**Per residue interaction decomposition**

To determine the contribution of each residue of the protein towards the binding of the drugs, the MM-GBSA method was used. MM-GBSA method decomposes the interaction energies for each residue by considering molecular mechanics and solvation energies without consideration of the contribution of entropies. Each residue contribution includes three terms: van der Waals contribution (ΔGvdw), electrostatic contribution (ΔGele) in a vacuum and solvation contribution (ΔGsolvation).

ΔG residue = ΔGvdw + ΔGele + ΔGsolvation

All energy components in the above equation were calculated using 5000 snapshots upon the stabilization of the complexes from the last 20ns of the MD simulation.

**Supplementary Results**

**SupplementaryTable S1. Interacting residues and the binding scores**

| **Compound name** | **Number of H-bonds** | | **H-bond forming residues** | **Number of hydrophobic interactions** | **Hydrophobic Interactions** | **Glide Score** | **X-score** |
| --- | --- | --- | --- | --- | --- | --- | --- |
| Madecassoside  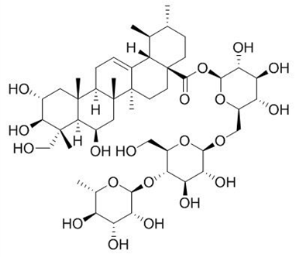 | 6 | Ser-83, Asn-86, Arg-135, Asn-154 | | 12 | His-82, Ser-84, Gly-85, His-87, Asp-132, Glu-136, His-152, Pro-153, Pro-232, Asp-238, Lys-241, Ser-242 | -12.58 kcal/mol | -10.33 kcal/mol |
| Madecassic acid  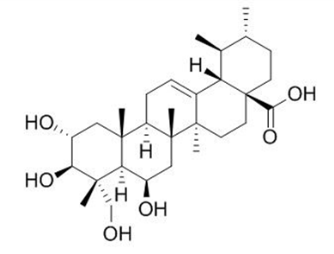 | 4 | | Asn-86, Arg-135, PLP-350 | 10 | His-82, Ser-83, Ser-84 , Gly-85, His-87, Asp-132, Pro-153, Asp-238, Gly-239, Ser-242 | -6.48 kcal/mol | -8.50 kcal/mol |
| Malonate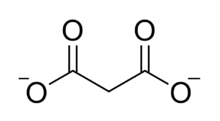 | 7 | | Ser-83, Ser-84, Asn-86, His-87, Arg-135, Ser-242 | 4 | Lys-56, Pro-153, Gly-239, PLP-350 | -5.96 kcal/mol | -6.12 kcal/mol |

**Binding free energy analysis by MM-GBSA**

Using the molecular dynamics trajectory of both madecassic acid and madecassoside the binding free energy calculations were carried out by molecular mechanics, the generalized born model commonly known as MM/GBSA. The MM/GBSA was carried out for the last 10 ns of the simulations using 5000 snapshots. The analysis suggested that upon stabilization of protein ligand complexes, both the ligands interacts with the active site residues of SR and remained bound with good binding free energy. Madecassoside showed slightly better binding free energy compared to madecassic acid which was evident from the analysis of MD simulation data. The detailed contribution of computation of average intramolecular energy (internal energy / enthalpy), corresponding entropies, solvation free energies of the solute along the MD trajectories of the free and bound protein and ligand is shown in Table S1. The distribution of binding free energies calculated by the different binding parameters (shown below) suggested that the binding of Madecassoside is driven by electrostatic interactions.

**Supplementary Table S2. Calculated total binding energies for serine racemase with madecassic acid and madecassoside by MM/GBSA.**

| **Energies** | **SR-Madecassic Acid**  **(kcal mol^-1^)** | **SR-Madecassoside**  **(kcal mol^-1^)** |
| --- | --- | --- |
| ΔE_VDW_ | - 45.60 | -76.69 |
| ΔE_ELE_ | 47.04 | -111.92 |
| ΔE_POL(GB)_ | -35.07 | 157.59 |
| ΔE_NPOL_ | -6.16 | -10.90 |
| ΔE_GAS_ | 1.43 | -188.62 |
| ΔE_SOLV_ | -41.24 | 146.69 |
| ΔG_TOT_ | **-39.80** | **-41.92** |

Absolute Free Energy ΔGTOT = (ΔEGAS + ΔESOLV)-TΔS; ΔEGAS + ΔESOLV = Enthalpy; TΔS = Solute Entropy; ΔEGAS = Total e energy of solute, ΔEGAS = ΔEVDW + ΔEELE; ΔEVDW = van der Waal’s energy; ΔEELE = Electrostatic/coulombic energy; ΔESOLV = Total energy of solvation; ΔESOLV = ΔEPOL(GB) + ΔENPOL; ΔEPOL(GB) = polar solvation contribution, generalized Born method; ΔENPOL = nonpolar contribution.

**Individual free energy contribution in ligand binding**

To understand the importance of the key interacting residues in the binding of ligands we calculated the per residue free energy decomposition. The key residues in binding of madecassic acid and madecassoside are listed in supplementary table 1 and the contribution of complete set of residues is shown in figure 6. In residues with binding free energy decomposed more than -1 kcal/mol are shown in the table. The most important residues predicted by MM-GBSA; contributing in the binding of both the ligands were positively charged, in case of medacassic acid Arg-135 is the highest contributor with -7.89 kcal/mol. Similarly, in madecassoside the most crucial residue predicted was Lys-241with -2.72 kcal/mol of free energy decomposed. Our calculations indicate that madecassic acid binds with few amino acids listed in table 2 that shows high free energy contribution compared to madecassoside. The common residues that are important for binding of both the ligands are Gly-85, Asn-86, Arg-135, Pro-153, Pro-232, Lys-241.


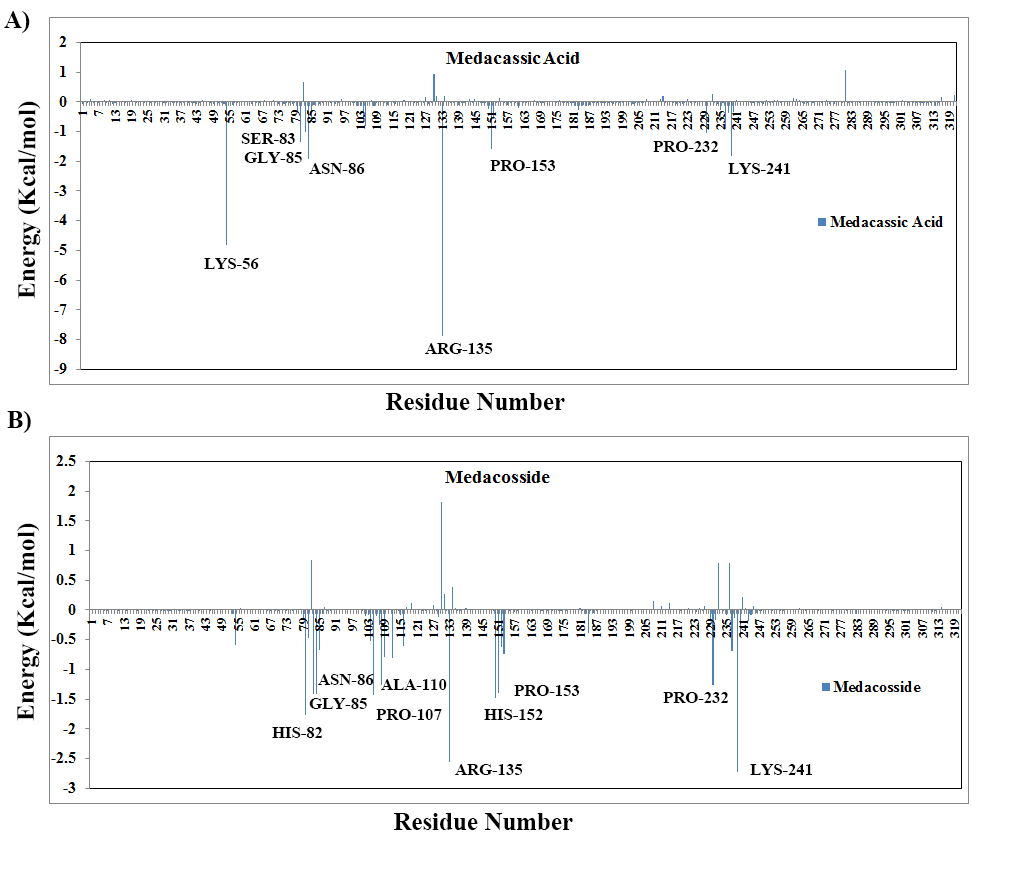


**Figure S5. Energy contribution for individual residues of Serine racemase in the binding of A) Madecassic acid and B) Madecassoside.**

**Supplementary Table S3. List of residues and their binding free energy decomposed in the binding of Madecassic Acid and Madecassoside.**

| **Residues** | **Total Energy Decomposition** | **Std. Dev.** | **Std. Err. of Mean** |
| --- | --- | --- | --- |
| **Medacassic Acid** | |  |  |
| ARG 135 | -7.89 | 1.41 | 0.08 |
| LYS 56 | -4.81 | 1.13 | 0.06 |
| ASN 86 | -1.91 | 0.60 | 0.03 |
| LYS 241 | -1.84 | 0.56 | 0.03 |
| PRO 153 | -1.58 | 0.30 | 0.02 |
| SER 83 | -1.37 | 0.65 | 0.04 |
| PRO 232 | -1.05 | 0.38 | 0.02 |
| GLY 85 | -1.02 | 0.38 | 0.02 |
| **Medacosside** | |  |  |
| LYS 241 | -2.72 | 0.67 | 0.03 |
| ARG 135 | -2.56 | 0.92 | 0.04 |
| HIS 82 | -1.77 | 0.51 | 0.02 |
| HIS 152 | -1.49 | 0.63 | 0.03 |
| PRO 107 | -1.43 | 0.24 | 0.01 |
| GLY 85 | -1.41 | 0.31 | 0.01 |
| ASN 86 | -1.41 | 1.19 | 0.05 |
| PRO 153 | -1.39 | 0.28 | 0.01 |
| PRO 232 | -1.27 | 0.29 | 0.01 |
| ALA 108 | -1.26 | 0.22 | 0.01 |

**Supplementary Table 4. Comparison of IC_50_ values of madecassoside with previously reported SR inhibitors.**

| LIGAND | | IC50 VALUE (µM) | REFERENCE |
| --- | --- | --- | --- |
|  | 2,2-Dichloromalonate | 57 | Vorlova et al., 2014 |
|  | Malonate | 67 | Vorlova et al., 2014 |
|  | 2-Hydroxymalonate | 94 | Vorlova et al., 2014 |
|  | Malonate | 131 | Mori et al., 2014 |
|  | 3-(3,5-Dibromophenyl)-N-(N0 -phenylacetylhydrazinocarbothioyl)acrylamide | 140 | Mori et al., 2017 |
|  | N-(4-benzyloxyphenyl)-2-(2-p-tolylethenesulfonylamino)acetamide | 270 | Mori et al., 2017 |
|  | N-{2-[3-(3,4-dichlorophenyl)ureido]phenyl}-4- methyl-benzenesulfonamide | 280 | Mori et al., 2017 |
|  | 1,2-cyclopropanedicarboxylate | 330 | Beato et al., 2015 |
|  | 2-Aminomalonate | 400 | Vorlova et al., 2014 |
|  | Malonate | 770 | Mori et al., 2017 |
|  | 2-Chloromalonate | 780 | Vorlova et al., 2014 |
|  | 2-Methylmalonate | 1850 | Vorlova et al., 2014 |
|  | 2,2-Difluoromalonate | 2160 | Vorlova et al., 2014 |
|  | 2,2-Dimethylmalonate | 5630 | Vorlova et al., 2014 |
|  | 2-Thiofenmalonate | 7030 | Vorlova et al., 2014 |
|  | 2,2-Bis(hydroxymethyl)malonate | 40,600 | Vorlova et al., 2014 |
|  | Madecassoside | 26 | Current study |

**SR Inhibition by Malonate**

To compare the inhibition potency of madecassoside and malonate, we have performed previously described chemiluminescent inhibition assay for malonate and IC50 value was calculated. To calculate the IC50 following concentrations of malonate were used, 0.1mM, 0.2mM, 0.3 mM, 0.5mM, 1.0 mM, 2.0 mM.

**Supplementary Figure S6: Inhibition assay of SR with malonate**

The calculated IC50 for malonate was 449.8 µM, which is 20 times higher than madecassoside. This proves that madecassoside is more potent inhibitor compare to malonate.

To confirm the specificity of madecassoside with SR, two other PLP dependent enzyme was used. Aspartate transaminase and Alanine transaminase both are PLP dependent transaminase enzymes. Estimation of Aspartate transaminase (AST) and Alanine transaminase (ALT) in the serum were done on Roche Hitachi Modular P 800 automated chemistry analyzer. Kits from Centronic GmbH (Cat No- GF04000060) were used for AST which are based on the IFCC principle The basic underlying principle is that the transamination reaction of α-Ketoglutarate and L-Aspartate (in the reagent) is catalyzed by AST (in serum) to form L-Glutamate and Oxaloacetate. The increase in oxaloacetate concentration is determined by an indicator reaction catalyzed by Malate dehydrogenase (in the reagent), wherein oxaloacetate reacts with NADH (in the reagent) to give L-Malate and NAD^+^. The reduction in NADH in the reagent is determined in a kinetic assay at 340 nm and 37^0^C.

Similarly for the estimation of Alanine transaminase (ALT), kits from Centronic (GF05000060) were used which are based on the IFCC principle. The basic underlying principle is that the transamination reaction of α-Ketoglutarate and L-Alanine (in the reagent) is catalyzed by ALT (in serum) to form L-Glutamate and Pyruvate. The increase in pyruvate concentration is determined by an indicator reaction catalyzed by Lactate dehydrogenase (in the reagent), wherein pyruvate reacts with NADH (in the reagent) to give L-Lactate and NAD^+^. The reduction in NADH in the reagent is determined in a kinetic assay at 340 nm and 37^0^C.

Two levels of controls, PreciControl ClinChem Multi 1 (normal level control) and PreciControl ClinChem Multi 2 (high level control) were run before starting the analysis. Both the controls were within the reference ranges provided by the manufacturer. The previous twenty run control standard deviation (SD) and coefficient of variation percentage (CV%) were both within acceptable limits. All the values are mentioned in Table 1.

| Table 1 | | | | | | |
| --- | --- | --- | --- | --- | --- | --- |
| Control | | Value | Target | Reference Range | SD | CV% |
| PreciControl ClinChem Multi 1 | AST (U/L) | 46 | 44.8 | 36.7-52.9 | 1.74 | 3.76 |
|  | ALT (U/L) | 46 | 46.5 | 38.1-54.9 | 1.42 | 3.09 |
| PreciControl ClinChem Multi 2 | AST (U/L) | 130 | 131 | 107-155 | 0.88 | 0.68 |
|  | ALT (U/L) | 113 | 117 | 96-138 | 2.28 | 2.02 |

To check the binding of madecassoside with AST and ALT, Serum from five donors was pooled to obtain a sufficient volume for analysis. Tests for AST and ALT were performed on the pooled serum which were used as baseline values for further analysis (Table 2). Aliquots of 150 µl of serum were made. 5 µl of distilled water (DW) was added to the first aliquot which served as the positive control (which would not inhibit the transaminase activity). The following concentration of madecassoside was added: 10 µM, 20 µM, 50 µM, 100 µM, 250 µM, 500 µM and 1000 µM respectively and reaction was incubated at 37°C for 1 hr. Tests for AST and ALT were run on the above-mentioned samples, the results of which are depicted in Table 2.

| Table 2 | | |
| --- | --- | --- |
| MADECASSOSIDE (µM) | ALT (U/L) | AST (U/L) |
| 0 | 133 | 79 |
| 10 | 134 | 77 |
| 50 | 132 | 77 |
| 100 | 131 | 78 |
| 250 | 133 | 77 |
| 500 | 129 | 76 |
| 1000 | 124 | 74 |

**Supplementary Figure S7: Activity Assay of Serine dehydratase and Aspartate racemase in the presence of madecassoside**

The following computational results confirm that madecassoside does not shows any significant binding with both the enzymes. That confirms the specificity of madecassoside for serine racemase.

**Table S5: Docking of 1P5J and 5HRC with Madecassoside and binding score**

| **No** | **Protein** | **Ligands** | **Glide score**  **kcal/mol** | **XSCORE**  **kcal/mol** | **MOE binding affinity**  **kcal/mol** |
| --- | --- | --- | --- | --- | --- |
| 1. | Serine dehydratse (1P5J) | Madecassoside | -3.75 | -3.90 | -3.59 |
| 2. | Aspartate racemase (5HRC) | Madecassoside | -3.41 | -3.27 | -3.10 |
